# Supplementary material for: Heat adaptation of phage T7 under an extended genetic code
Source: Virus Evol. 2021 Dec 1;7(2):veab100. doi: 10.1093/ve/veab100 (PMC8923235; doi:10.1093/ve/veab100)
Supplement: veab100_Supp [file veab100_supp.zip › Sequencing_Analysis.zip!/Sequencing_Analysis/A2/evidence/SNP_15.html]

BRESEQ :: Evidence


| Predicted mutation | | | | | | |
| --- | --- | --- | --- | --- | --- | --- |
| evidence | seq id | position | mutation | annotation | gene | description |
| RA | NC\_001604.1 | 37,346 | T→C | intergenic (–/–) | – / – | –/– |

| Read alignment evidence... | | | | | | | | | | | |
| --- | --- | --- | --- | --- | --- | --- | --- | --- | --- | --- | --- |
|  | seq id | position | | ref | new | freq | score (cons/poly) | reads | annotation | genes | product |
| \* | NC\_001604.1 | 37,346 | 0 | T | C | 99.5% | 1343.2 / ‑4.0 | 441 | intergenic (–/–) | *–/–* | –/– |
| Reads supporting (aligned to +/- strand):  **ref** base T (0/2);  **new** base C (209/228);  **total** (210/231) | | | | | | | | | | | |
| Fisher's exact test for biased strand distribution *p*-value = 5.00e-01 | | | | | | | | | | | |
| Kolmogorov-Smirnov test that lower quality scores support variant *p*-value = 9.16e-01 | | | | | | | | | | | |

Only 100 of 441 total aligned reads displayed.  

`AGTTGAGGCTGCGAAGAGCACTCAAAGAGCAATCGATGCGGTATCTGCTAAGTATCAAGAAGACCTTGCCGCGCTGGAAGGGAGCACTGATAGGATTATTTCTGATTTGCGTAGCGACAATAAGCGGTTGCGCGTCAGAGTCAAAACTACCGGAACCTCCGATGGTCAGTGTGGATTCGAGCCTGATGGTCGAGCCGAACTTGACGACCGAGATGCTAAACGTATTCTCGCAGTGACCCAGAAGGGTGACGCATGGATTCGTGCGTTACAGGATACTATTCGTGAAC‑TGCAACGTAAGTAGGAAATCAAGTAAGGAGGCAATGTGTCTACTCAATCCAAT‑CGTAATGCGCTCGTA‑GTGGCGCAACTGAAAGGAGACTTC‑GTGGCGTTCCTATTCGTCTTATGGAAGGCGCTAAACCTACCGGTGCCCAC‑TAAGTGTCAGATTGACATGGCTAAGGTGCTGGCGAAT‑GGAGACAACAAGAAGTTCATCTTACAGGCTTTCCGTGGTATCGGTAAGTCGTTCATCACATGTGCGTTCGTTGTGTGGTCCTTATGGAGAGACCCTCAGTTGAAGATACTTATCGTATCAGCCTCTAAGG  >  NC_001604.1/37048‑37643`  

                                                                                                                                                                                                                                                                                                           `|`                                                                                                                                                                                                                                                                                                                
 `gTTGAGGCTGCGAAGAGCACTCAAAGAGCAATCGATGCGGTATCTGCTAAGTATCAAGAAGACCTTGCCGCGCTGGAATGGAGCACTGATAGGATTATTTCTGATTTGCGTAGCGACAATAAGCGGTTGCGCGTCAGAGTCAAAACTACCGGAACCTACGATGGTCAGTGTGGATTCGAGCCTGATGGTCGAGCCGAACTTGACGACCGAGATGCTACACGTATTCTCGCAGTGACCCAGAAGGGTGACGCATGGATTCGTGCGTTACAGGATACTATTCGTGAAC‑TGCAACGTAAGCAgg                                                                                                                                                                                                                                                                                                             >  1:507732/1‑301 (MQ=255)`  
 `gTTGAGGCTGCGAAGAGCACTCAAAGAGCAATCGATGCGGTATCTGCTAAGTATCAAGAAGACCTTGCCGCGCTGGAAGGGAGCACTGATAGGATTATTTCTGATTTGCGTAGCGACAATAAGCGGTTGCGCGTCAGAGTCAAAACTACCGGAACCTCCGATGGTCAGTGTGGATTCGAGCCTGATGGTCGAGCCGAACTTGACGACCGAGATGCTAAACGTATTCTCGCAGTGACCCAGAAGGGTGACGCATGGATTCGTGCGTTACAGGATACTATTCGTGAAC‑TGCAACGTAAGCAgg                                                                                                                                                                                                                                                                                                             <  1:681162/301‑1 (MQ=255)`  
                    `cTCAAAGAGCAATCGATGCGGTATCTGCTAAGTATCAAGAAGACCTTGCCGCGCTGGAAGGGAGCACTGATAGGATTATTTCTGATTTGCGTAGCGACAATAAGCGGTTGCGCGTCATAGTCAAAACTACCGGAACCTCCGATGGTCAGTGTGGATTCGAGCCTGATGGTCGAGCCGAACTTGACGACCGAGATGCTAAACGTATTCTCGCAGTGACCCAGAAGGGTGACGCATGGATTCGTGCGTTACAGGATACTATTCGTGAAC‑CGCAACGTTAGCAGGAAATCAAGTAAGGAGTCaa                                                                                                                                                                                                                                                                                          >  1:361305/1‑301 (MQ=255)`  
                      `cAAAGAGCAATCGATGCGGTATCTGCTAAGTATCAAGAAGACCTTGCCGCGCTGGAAGGGAGCACTGATAGGATTATTTCTGATTTGCGTAGCGACAATAAGCGGTTGCGCGTCAGAGTCAAAACTACCGGAACCTCCGATGGTCAGTGTGGATTCGAGCCTGATGGTCGAGCCGAACTTGACGACCGAGATGCTAAACGTATTCTCGCAGTGACCCAGAAGGGTGACGCATGGATTCGTGCGTTACAGGATACTATTCGTGAAC‑TGCAACGTAAGCAGGAAATCAAGTAAGGAGGCAAtg                                                                                                                                                                                                                                                                                        >  1:643087/1‑301 (MQ=255)`  
                             `aaaTCGATGCGGTATCTGCTAAGTATCAAGAAGACCTTGCCGCGCTGGAAGGGAGCACTGATAGGATTATTTCTGATTTGCGTAGCGACAATAAGCGGTTGCGCGTCAGAGTCAAAACTACCGGAACCTCCGATGGTCAGTGTGGATTCGAGCCTGATGGTCGAGCCGAACTTGACGACCGAGATGCTAAACGTATTCTCGCAGTGACCCAGAAGGGTGACGCATGGATTCGTGCGTTACAGGATACTATTCGTGAAC‑TGCAACGTAAGCAGGAAATCAAGTAAGGAGGCAATGTGTCTAc                                                                                                                                                                                                                                                                                 <  1:158810/300‑1 (MQ=255)`  
                               `gtgtataagagacaggCTAAGTATCAAGAAGACCTTGCCGCGCTGGAAGGGAGCACTGATAGGATTATTTCTGATTTGCGTAGCGACAATAAGCGGTTGCGCGTCAGAGTCAAAACTACCGGAACCTCCGATGGTCAGTGTGGATTCGAGCCTGATGGTCGAGCCGAACTTGACGACCGAGATGCTAAACGTATTCTCGCAGTGACCCAGAAGGGTGACGCATGGATTCGTGCGTTACAGGATACTATTCGTGAAC‑TGCAACGTAAGCAGGAAATCAAGTAAGGAGGCAATGTGTCTACTc                                                                                                                                                                                                                                                                               <  1:161842/286‑1 (MQ=255)`  
                               `atagATGCTGTATCTGCTAAGTATCAAAGAGACCTTGCCGAGCTGGAAGGGAGCACGGATAGGATTATTGCTGATATGCGTAGCGACCATAAGCGGTTGCGAGTCAAAGTCAAAACTACCGGAACCGCCGATGGTCAGTGTGGATTCGAGCCTGATGGTCGAGCCGAACTTGACGACCGAGATGCTAAACGTATTCTCGCAGTGACACAGAAGGGTGACGCATGGATTCGTGCGTTACAGGATACTATTCGTGAAC‑TGCAACGTAAGCAGGAAATCAAGTAAGGAGGCAATGGGTCTACTc                                                                                                                                                                                                                                                                               <  1:151943/298‑1 (MQ=255)`  
                               `acagATGCGGTATCTGCTAAGTATCAAGAAGACCTTGCCGCGCTGGAAGGGAGCACTGATAGGATTATTTCTGATTTGCGTAGCGACGATAAGCGGTTGCGCGTCAGAGTCAAAACTACCGGAACCTCCGATGGTCAGTGTGGATTCGAGCCTGATGGTCGAGCCGAACTTGACGACCGAGATGCTAAACGTATTCTCGCAGTGACCCAGAAGGGTGACGCATGGATTCGTGCGTTACAGGATACTATTCGTGAAC‑TGCAACGTAAGCAGGAAATCAAGTAAGGAGGCAATGTGTCTACTc                                                                                                                                                                                                                                                                               <  1:762548/298‑1 (MQ=255)`  
                               `aatctgtatacgaggcagcattATCAAGAAGGCCTTGCCGCGCTGTAAGGGAGCACTGATAAGATTATTTCTGATTTGCGTTGCTACCATAAGCGGTTGCGCGTCAGATTCAAAACTACCGGAACCTCCGATGGTCAGTGTGGATTCGAGCCTGATGGTCGAGCCGAACTTGACGACCGAGATGCTAAACGTATTCTCGCAGTGACCCAGAAGGGTGACGCATGGATTCGTGCGTTACAGGATACTATTCGTGAAC‑TGCAACGTAAGCAGGAAATCAAGTAAGGAGGCAATGTGTCTACTc                                                                                                                                                                                                                                                                               <  1:310888/280‑1 (MQ=255)`  
                               `aTCGATGCGGTATCTGCTAAGTATCAAGAAGACCTTGCCGCGCTGGAAGGAAGCACTGATAGGATTATTTCTGATTTGCGTAGCGACAATAAGCGGTTGCGCGTCAGAGTCAAAACTACCGGAACCTCCGATGGTCAGTGTGGATTCGAGCCTGATGGTCGAGCCGAACTTGACGACCGAGATGCTAAACGTATTCTCGCAGTGACCCAGAAGGGTGACGCATTGATTCGTGCGTTACAGGATACTATTCGTGAAC‑TGCAACGTAAGCAGGAAATCAAGtctgtctcttatacacatctcc                                                                                                                                                                                                                                                                               >  1:100685/1‑280 (MQ=255)`  
                                    `tGCGGTATCTGCTAAGTATCAAGAAGACCTTGCCGCGCTGGAAGGGAGCACTGATAGGATTATTTCTGATTTGCGTAGCGACAATAAGCGGTTGCGCGTCAGAGTCAAAACTACCGGAACCTCCGATGGTCAGTGTGGATTCGAGCCTGATGGTCGAGCCGAACTTGACGACCGAGATGCTAAACGTATTCTCGCAGTGACCCAGAAGGGTGACGCATGGATTCGTGCGTTACAGGATACTATTCGTGAAC‑TGCAACGTAAGCAGGAAATCAAGTAAGGAGGCAATGTGTCTACTcaatcc                                                                                                                                                                                                                                                                          <  1:303563/301‑1 (MQ=255)`  
                                    `ggagatgtgtataagagacagagaagaCCTTGCCGCGCTGGAAGGGAGCACTGATAGGATTATTTCTGATTTGCGTAGCGACAATAAGCGGTTGCGCGTCAGAGTCAAAACTACCGGAACCTCCTATGGTCAGTGTGGATTCGAGCCTGATGGTCGAGCCGAACTTGACTACCGAGATGCTAAACGTATTCTCGCAGTGACCCAGAAGGGTGACGCATGGATTCGTGCGTTACAGGATACTATTCGTGAAC‑TGCAACGTAAGCAGGAAATCAAGTAAGGAGGCAATGTGTCTACTcaatcc                                                                                                                                                                                                                                                                          <  1:124715/280‑1 (MQ=255)`  
                                        `gTATCTGCTAAGTATCAAGAAGACCTTGCCGCGCTGGAAGGGAGCACTGATAGGATTATTTCTGATTTGCGTAGCGACAATAAGCGGTTGCGCGTCAGAGTCAAAACTACCGGAACCTCCGATGGTCAGTGTGGATTCGAGCCTGATGGTCGAGCCGAACTTGACGACCGAGATGCTAAACGTATTCTTGCAGTGACCCAGAAGGGTGACGCATGGATTCGTGCGTTACAGTATACTATTCGTGAAC‑TGCAACGTAAGCAGGAAATCAAGTAAGGAGGCAATGTGTCTACTcaatcccat‑c                                                                                                                                                                                                                                                                     >  1:499083/1‑301 (MQ=255)`  
                                        `gTATCTGCTAAGTATCAAGAAGACCTTGCCGCGCTGGAAGGGAGCACTGATAGGATTATTTCTGATTTGCGTAGCGACAATAAGCGGTTGCGCGTCAGAGTCAAAACTACCGGAACCTCCGATGGTCAGTGTGGATTCGAGCCTGATGGTCGAGCCGAACTTGACGACCGAGATGCTAAACGTATTCTCGCAGTGACCCAGAAGGGTGACGCATGGATTCGTGCGTTACAGGATACTATTCGTGAAC‑TGCAACGTAAGCAGGAAATCAAGTAAGGAGGCAATGTGTCTACTcatcccctgt                                                                                                                                                                                                                                                                      >  1:421838/1‑293 (MQ=255)`  
                                         `tATCTGCTAAGTATCAAGAAGACCTTGCCGCGCTGGAAGGGAGCACTGATAGGATTATTTCTGATTTGCGTAGCGACAATAAGCGGTTGCGCGTCAGAGTCAAAACTACCGGAACCTCCGATGGTCAGTGTGGATTCGAGCCTGATGGTCGAGCCGAACTTGACGACCGAGATGCTAAACGTATTCTCGCAGTGACCCAGAAGGGTGACGCATGGATTCGTGCGTTACAGGATACTATTCGTGAAC‑TGCAACGTAAGCAGGAAATCAAGTAAGGAGGCAATGTGTCTACTCAATCCAAT‑Cg                                                                                                                                                                                                                                                                    <  1:479853/301‑1 (MQ=255)`  
                                          `aTCTGCTAAGTATCAAGAAGACCTTGCCGCGCTGGAAGGGAGCACTGATAGGATTATTTCTGATTTGCGTAGCGACAATAAGCGGTTGCGCGTCAGAGTCAAAACTACCGGAACCTCCGATGGTCAGTGTGGATTCGAGCCTGATGGTCGAGCCGAACTTGACGACCGAGATGCTAAACGTATTCTCGCAGTGACCCAGAAGGGTGACGCATGGATTCTTGCGTTACAGGATACTATTCGTGAAC‑TGCAACGTAAGCAGGACATCAAGTAAGGAGGCAATGTGTCTACTCAATCCAAT‑CGt                                                                                                                                                                                                                                                                   >  1:813732/1‑301 (MQ=255)`  
                                              `gCTAAGTATCAAGAAGACCTTGCCGCGCTGGAAGGGAGCACAGATAGGATTATTTCTGATTTGCGTAGCGACAATAAGCGGTTGCGCGTCAGAGTCAAAACTACCGGAACCTCCGATGGTCAGTGTGGATTCGAGCCTGATGGTCGAGCCGAACTTGACGACCGAGATGCTAAACGTATTCTCGCAGTGACCCAGAAGGGTGACGCATGGATTCGTGCGTTACAGGATACTATTCGTGAAC‑TGCAACGTAAGCAGGAAATCAAGTAAGGAGGCAATGTGTATACTCAATCCAAT‑CGTCAtt                                                                                                                                                                                                                                                               >  1:523535/1‑300 (MQ=255)`  
                                                         `tgtataagagacagcgcTGGAAGGTAGCACTGATAGGATTATTTCTGATTTGCGTAGCGACAATAAGCGGTTGCGCGTCAGAGTCAAAACTACCGGAACCTCCGATGGTCAGTGTGGATTCGAGCCTGATGGTCGAGCCGAACTTGACGACCGAGATGCTAAACGTATTCTCGCAGTGACCCAGAAGGGTGACGCATGGATTCGTGCGTTACAGGATACTATTCGTGAAC‑TGCAACGTAAGCAGGAAATCAAGTAAGGAGGCAATGTGTCTACTCAATCCAAT‑CGTAATGCGCTCGTA‑GTg                                                                                                                                                                                                                                                   <  1:474775/288‑1 (MQ=255)`  
                                                          `tcggagatgtgtataagagacaggAGCACTGATAGGATTATTTCTGATTTGCGTAGCGACAATAAGCGGTTGCGCGTCAGAGTCAAAACTACCGGAACCTCCGATGGTCAGTGTGGATTCGAGCCTGATGGTCGAGCCGAACTTGACGACCGAGATGCTAAACGTATTCTCGCAGTGACCCAGAAGGGTGACGCATGGATTCGTGCGTTACAGGATACTATTCGTGAAC‑TGCAACGTAAGCAGGAAATCAAGTAAGGAGGCAATGTGTCTACTCAATCCAAT‑CGTAATGCGCTAGTA‑GTgg                                                                                                                                                                                                                                                  <  1:382578/279‑1 (MQ=255)`  
                                                                       `gacagccaGGGAGCACTGATAGGATTATTTCTGATTTGCGTATCGACAATAAGCGGTTGCGCGTCAGAGTCAAAACTACCGGAACATCCGATGGTCAGTGTGGATTCGAGCCTGATGGTCGAGCCGAACTTGACGACCGAGATGCTAAACGTATTCTCGCAGTGACCCAGAAGGGTGACGCATGGATTCGTGCGTTACAGGATACTATTCGTGAAC‑TGCAACGTAAGCATGAAATCAAGTAAGGAGGCAATGTGTCTACTCAATCCAAT‑CGTAATGCGCTCGTA‑GTGGCGCAACTGAAAgg                                                                                                                                                                                                                                     <  1:807493/294‑1 (MQ=255)`  
                                                                         `cTGGAAGGGAGCACTGATAGGATTATTTCTGATTTGCGTAGCGACAATAAGCGGTTGCGCGTCAGAGTCAAAACTACCGGAACCTCCGATGGTCAGTGTGGATTCGAGCCTGATGGTCGAGCCGAACTTGACGACCGAGATGCTAAACGTATTCTCGCAGTGACCCAGAAGGGTGACGCATGGATTCGTGCGTTACATGATACTATTCGTGAAC‑TGCAACGTAAGCAGGAAATCAAGTAAGGAGGCAATGTGTCTACTCAATCCAAT‑CGTAATGCGCTCGTA‑GTGGCGCAACTGAAAGgag                                                                                                                                                                                                                                   >  1:312313/1‑301 (MQ=255)`  
                                                                             `aaGGGAGCACTGATAGGATTATTTCTGATTTGCGTAGCGACAATAAGCGGTTGCGCGTCAGAGTCAAAACTACCGGAACCTCCGATGGTCAGTGTGGATTCTAGCCTGATGGTCGAGCCGAACTTGACGACCGAGATGCTAAACGTATTCTTGCAGTGACCCAGAAGGGTGACGCATGGATTCGTGCGTTACAGGATACTATTCGTGAAC‑TGCAACGTAAGCAGGAAATCAAGTAAGGAGGCAATGTGTCTACTCAATCCAAT‑CGTAATGCGCTCGTA‑GTGGCGCAACtctgtctcttata                                                                                                                                                                                                                               >  1:253314/1‑289 (MQ=255)`  
                                                                              `aGGGAGCACTGATAGGATTATTTCTGATTTGCGTAGCGACAATAAGCGGTTGCGCGTCAGAGTCAAAACTACCGGAACCTCCGATGGTCAGTGTGGATTCGAGCCTGATGGTCGAGCCGAACTTGACGACCGAGATGCTAAACGTATTCTCGCAGTGACCCAGAAGGGTGACGCATGGATTCGTGCGTTACAGGATACTATTCGTGAAC‑TGCAACGTAAGCAGGAAATCAAGTAAGGAGGCAATGTGTCTACTCAATCCAAT‑CGTAATGCGCTCGTA‑GTGGCGCAACTGAAAGGAGACTTc                                                                                                                                                                                                                              >  1:613026/1‑301 (MQ=255)`  
                                                                                `ggAGCACTGATAGGATTATTTCTGATTTGCGTAGCGACAATAAGCGGTTGCGCGTCAGAGTCAAAACTACCGGAACCTCCGATGGTCAGTGTGGATTCGAGCCTGATGGTCGAGCCGAACTTGACGACCGAGATGCTAAACGTATTCTCGCAGTGACCCAGAAGGGTGACGCATGGATTCGTGCGTTACAGGATACTATTCGTGAAC‑TGCAACGTAAGCAGGAAATCAAGTAAGGAGGCAATGTGTCTACTCAATCCAAT‑CGTAATGCGCCCGTA‑Gtctgtctcttatacacatctccgag                                                                                                                                                                                                                            >  1:297982/1‑277 (MQ=255)`  
                                                                                   `gCACTGATAGGATTATTTCTGATTTGCGTAGCGACAATAAGCGGTTGCGCGTCAGAGTCAAAACTACCGGAACCTCCGATGGTCAGTGTGGATTCGAGCCTGATGGTCGAGCCGAACTTGACGACCGAGATGCTAAACGTATTCTCGCAGTGACCCAGAAGGGTGACGCATGGATTCGTGCGTTACAGGATACTATTCGTGAAC‑TGCAACGTAAGCAGGAAATCAAGTAAGGAGGCAATGTGTCTACTCAATCCAAT‑CGTAATGCGCTCGTA‑GTAGCGCAACTGAAAGGAGACTTC‑GTGGc                                                                                                                                                                                                                        <  1:181542/301‑1 (MQ=255)`  
                                                                                    `aaCTGATAGGATTATTTCTGATTTGCGTAGCGACAATAAGCGGTTGCGCGTCAGAGTCAAAACTACCGGAACCTCCGATGGTAAGTGTGGATTCGAGCCTGATGGTCGAGCCGAACTTGACGACCGAGATGCTAAACGTATTCTCGCAGTGACCCAGAAGGGTGACGCATGGATTCGTGCGTTACAGGATACTATTCGTGAAC‑TGCAACGTAAGCAGGAAATCAAGTAAGGAGGCAATGTGTCTACTCAATCCAAT‑CGTAATGCGCTCGTA‑GTGGCGCAACTGAAAGGAGACTTC‑GTGGCg                                                                                                                                                                                                                       <  1:548179/300‑1 (MQ=255)`  
                                                                                      `cTGATAGGATTATTTCTGATTTGCGTAGCGACAATAAGCGGTTGCGCGTCAGAGTCAAAACTACCGGAACCTCCGATGGTCAGTGTGGATTCGAGCCTGATGGTCGAGCCGAACTTGACGACCGAGATGATAAACATATTCTCGCAGTGACCCAGAAGGGTGACGCATGGATTCGTGCGTTACAGGATACTATTCGTGAAC‑TGCAACGTAAGCAGGAAATCAAGTAAGGAGGCAATGTGTCTACTCAATCCAAT‑CGTAATTCGCTCGTA‑GTGGCGCAACTGAAAGGAGACTTC‑GTGGctgt                                                                                                                                                                                                                     >  1:133973/1‑298 (MQ=255)`  
                                                                                             `gATTATTTCTGATTTGCGTAGCGACAATAAGCGGTTGCGCGTCAGAGTCAAAACTACCGGAACCTCCGATGGTCAGTGTGGATTCGAGCCTGATGGTCGAGCCGAACTTGACGACCGAGATGCTAAACGTATTCTCGCAGTGACCCAGAAGGGTGACGCATGGATTCGTTCGTTACATGATACTATTCGTGAAC‑TGCAACGTAAGCAGGAAATCAAGTAAGGAGGCAATGTGTCTACTCAATCCAAT‑CGTAATGCGCTCGTA‑GTGGCGCAACTGAAAGGAGACTTC‑GTGGCGTTCCTAttt                                                                                                                                                                                                              >  1:13622/1‑300 (MQ=255)`  
                                                                                                  `tttCTGATTTGCGTAGCGACAATAAGCGGTTGCGCGTCAGAGTCAAAACTACCGGAACCTCCGATTGTCAGTGTGGATTCGAGCCTGATGGTCGAGCCGAACTTGACGACCGAGATGCTAAACGTATTCTCGCAGTGACCCAGAAGGGTGACGCATGGATTCGTGCGTTACAGGATACTATTCGTGAAC‑TGCAACGTAAGCAGGAAATCAAGTAAGGAGGCAATGTGTCTACTCAATCCAAT‑CGTAATGCGCTCGTA‑GTGGCGCAACTGAAAggctgtctcttatacacatctccgagcac                                                                                                                                                                                                          >  1:433957/1‑274 (MQ=255)`  
                                                                                                      `tggtttGCGTAGCGACAATAAGCGGTTGCGCGTCAGAGTCAAAACTACCGGAACCTCCGATGGTCAGTGTGGATTCGAGCCTGATGGACGAGCCGAACTTGACGACCGAGATGCTAAACGTATTCTCGCAGTGACCCAGAAGGGTGACGCATGGATTCGTGCGTTACAGGATACTATTCGTGAAC‑TGCAACGTAAGCAGGAAATCAAGTAAGGAGGCAATGTGTCTACTCAATCCAAT‑CGTAATGCGCTCGTA‑GTGGCGCAACTGAAAGGAGACTTC‑GTGGCGTTCATATTCGTCTTATgg                                                                                                                                                                                                     <  1:582906/298‑1 (MQ=255)`  
                                                                                                      `tGATTTGCGTAGCGACAATAAGCGGTTGCGCGTCAGAGTCAAAACTACCGGAACCTCCGATGGTCAGTGTGGATTAGAGCCTGATGGTCGAGCCGAACTTGACGACCGAGATGCTAAACGTATTCTCGAAGTGACCCAGAAGGGTGACGCATGGATTGGTGCATTACAGGATACTATTCGTGAAC‑TGCAACGTAAGCAGGAAATCAAGTAAGGAGGCAATGTGTCTACTCAATCCAAT‑CGTAATGCGCTCGTA‑GTGGCGCAACTGAAAGGAGACTTC‑GTGGCGTTCCTATTCGTCTTATgg                                                                                                                                                                                                     <  1:31409/301‑1 (MQ=255)`  
                                                                                                      `agatgggtataagagacagaaGCGGTTGCGCGTCAGAGTCAAAACTACCGGAACCTCCGATGGTCAGTGTGGATTCTAGCCTGATGGTCGAGCCGAACTTGACGACAGAGATGCTAAACGTATTCTCGCAGAGACCCAGAAGGGTGACGCATGGATTCGTGCGTTACAGGATACTATTCGTGAAC‑TGCAACGTAAGCAGGAAATCAAGTAAGGAGGCAATGTGTCTACTCAATCCAAT‑CGTAATGCGCTCGTA‑GTGGCGCAACTGAAAGGAGACTTC‑GTGGCGTTCCTATTCGTCTTATgg                                                                                                                                                                                                     <  1:794026/282‑1 (MQ=255)`  
                                                                                                       `gtataagagacagACAATAAGCGGTTGCGCGTCAGAGTCAAAACTACCGGAACCTCCGATGGTCAGTGTGGATTCGAGCCTGATGGTCGAGCCGAACTTGACGACCGAGATGCTAAACGTATTCTCGCAGTGACCCAGAAGGGTGACGCATGGATTCGTGCGTTACAGGATACTATTCGTGAAC‑TGCAACGTAAGCAGGAAATCAAGTAAGGAGGCAATGTGTCTACTCAATCCAAT‑CGTAATGCGCTCGTA‑GTGGCGAAACTGAAAGGAGACTTC‑ATGGCGTTCCTATTCGTCTTATGGa                                                                                                                                                                                                    <  1:54163/289‑1 (MQ=255)`  
                                                                                                          `ataagagacagcAATAAGCGGTTGCGCTTCAGAGTCAAAACTACCGGAACCTCCGATGGTCAGTGTGGATTCGAGCCTGATGGTCGAGCCGAACTTGACGACCGAGATGCTAAACGTATTCTCGCAGTGACCCAGAAGGGTTACGCATGGATTCGTGCGTTACAGGATACTATTCGTGAAC‑TGCAACGTAAGCAGGAAATCAAGTAAGGAGGCAATGTGTCTACTCAATCCAAT‑CGTAATGCGCTCGTA‑GTGGCGCAACTGAAAGGAGACTTC‑GTGGCGTTCCTATTCGTCTTATGAAAgg                                                                                                                                                                                                 <  1:714364/290‑1 (MQ=255)`  
                                                                                                                    `ggagatgtgtataagagacagggggcAAAACTACCGTAACCTCCGATGGTCAGTGTGGATTCGAGCCTGATGGTCGAGCCGAACTTGACGACCGAGATGCTAAACGTATTCTCGCAGTGACCCAGAAGGGTGACGCATGGATTCGTGCGTTACAGGATACTATTCGTGAAC‑TGCAACGTAAGCAGGAAATCAAGTAAGGAGGCAATGTGTCTACTCAATCCAAT‑CGTAATGCGCTCGTA‑GTGGCGCAACTGAAAGGAGACTTC‑GTGGCGTTCCTATTCGTCTTATGGAAGGCGCTAAACCt                                                                                                                                                                                       <  1:1885/276‑1 (MQ=255)`  
                                                                                                                    `aCAATAAGCGGTTGCGCGTCAGAGTCAAAACTACCGGAACCTCCGATGGTCAGTGTGGATTCGAGCCTGATGGTCGAGCCGAACTTGACGACCGAGATGCTAAACGTATTCTCGCAGTGACCCAGAAGGGTGACGCATGGATTCGTGCGTTACAGGATACTATTCGTGAAC‑TGCAACGTAAGCAGGAAATCAAGTAAGGAGGCAATGTGTCTACTCAATCCAAT‑CGTAATGCGCTCGTA‑GTGGCGCAACTGAAAGTAGACTTC‑GTGGCGTTCCTATTCGTCTTATGGAAGGCGCTAAACCt                                                                                                                                                                                       >  1:623159/1‑301 (MQ=255)`  
                                                                                                                         `aggCGGTTGCGCGTCAGAGGCAAAACTACCGGAACCTCCGATGGTCAGTGTGGATTCGAGCCTGATGGTCGAGCCGAACTTGACGACCTAGATGCTAAACGTATTCTCGCAGTGACCCAGAAGGGTGACGCATGGATTCGTGCGTTACAGGATACTATTCGTGAAC‑TGCAACGTAAGCAGGAAATCAAGTAAGGAGGCAATGTGTCTACTCAATCCAAT‑CGTAATGCGCTCGTA‑GTGGCGCAACTGAAAGGAGACTTC‑GTGGCGTTCCTATTCGTCTTATGGAAGGCGCTAAACCTACCgg                                                                                                                                                                                  <  1:46166/299‑1 (MQ=255)`  
                                                                                                                         `aaGCGGTTGCGCGTCAGAGTCAAAACTACCGGAACTTCCGATGGTCAGTGTGGATTCGAGCCTGATGGTCGAGCCGAACTTGACGACCGAGATGCTAAACGTATTCTCGCAGTGACCCAGAAGTGTGACGCATGGATTCGTGCGTTACAGGATACTATTCGTGAAC‑TGCAACGTAAGCAGGAAATCAAGTAAGGAGGCAATGTGTCTACTCAATCCAAT‑CGTAATGCGCTCGTA‑GTGGCGCAACTGAAAGGAGACTTC‑GTGGCGTTCCTATTCGTCTTATGGAAGGCGCTAAACCTACCgg                                                                                                                                                                                  >  1:557268/1‑301 (MQ=255)`  
                                                                                                                            `cGGTTGCGCGTCAGAGTCAAAACTACCGGAACCTCCGATGGTCAGTGTGGATTCGAGCCTGATGGTCGAGCCGAACTTGACGACCTAGATGCTAAACGTATTCTCGCAGTGACCCAGAAGGGTGACGCATGGATTCGTGCGTTACAGGATACTATTCGTGAAC‑TGCAACGTAAGCAGGAAATCAAGTAAGGAGGCAATGTGTCTACTCAATCCAAT‑CGTAATGCGCTCGTA‑GTGGCGCAACTGAAAGGAGACTTC‑GTGGCGTTCCTATTCGTCTTATGGAAGGCGCTAAACCTACCGGTGc                                                                                                                                                                               >  1:568834/1‑301 (MQ=255)`  
                                                                                                                                 `tcgcgTCAGAGTCAAAACTACCGGAACCTCCGATGGTCAGTGTGGATTCGAGCCTGATGGTCGAGCCGAACTTGACGACCGAGATGCTAAACGTATTCTCGCAGTGACCCAGAAGGGTGACGCATGGATTCGTGCGTTACAGGATACTATTCGTGAAC‑TGCAACGTAAGCAGGAAATCAAGTAAGGAGGCAATGTGTCTACTCAATCCAAT‑CGTAATGCGCTCGTA‑GTGGCGCAACTGAAAGGAGACTTC‑GTGGCGTTCCTATTCGTCTTATGGAAGGCGCTAAACCTACCGGTGCCCAC‑t                                                                                                                                                                         <  1:779381/300‑1 (MQ=255)`  
                                                                                                                                            `agagacagaCCTGAACCTCCGATGGTCAGTGTGGATTCGAGCCTGATGGTCGAGCCGAACTTGACGACCGAGATGCTAAACGTATTCTCGCAGTGACCCAGAAGGGTGACGCATGGATTCGTGCGTTACAGGATACTATTCGTGAAC‑TGCAACGTAAGCAGGAAATCAAGTAAGGAGGCAATGTGTCTACTCAATCCAAT‑CGTAATGCGCTCGTA‑GTGGCGCAACTGAAAGGAGACTTC‑GTGGCGTTCCTATTCGTCTTATGGAAGGCGCTAAACCTACCGGTGCCCAC‑TAAGTGTCAGAt                                                                                                                                                              <  1:147572/293‑1 (MQ=255)`  
                                                                                                                                                 `gcTACCGGAACCTCCGATGGTCAGTGTGGATTCGAGCCTGATTGTCGAGCCGAACTTGACGACCGAGATGCTAAACGTATTCTCGCAGTGACCCAGAAGGGTGACGCATGGATTCGTGTGTTACAGGATACTATTCGTGAAC‑TGCAACGTAAGCAGGAAATCAAGTAAGGAGGCAATGTGTCTACTCAATCCAAT‑CGTAATGCGCTCGTA‑GTGGCGCAACTGAAAGGAGACTTC‑GTTGCGTTCCTATTCGTCTTATGGAAGGCGCTAAACCTACCGGTGCCCAC‑TAAGTGTCAGATTGACa                                                                                                                                                         <  1:554011/300‑1 (MQ=255)`  
                                                                                                                                                    `aCCGGAACCTCCGATGGTCAGTGTGGATTCGAGCCTGATGGTCGAGCCGAACTTGACGACCGAGATGCTAAACGTATTCTCGCAGTGACCCAGAATGGTGACGCATGGATTCGTGCGTTACAGGATACTATTCGTGAAC‑TGCAACGTAAGCAGGAAATCAAGTAAGGAGGCAATGTGTCTACTCAATCCAAT‑CGTAATTCGCTCGTA‑GTGGCGCAACTGAAAGGAGACTTC‑GTGGCGTTCCTATTCATCTTATGGAAGGCGCTAAACCTACCGTTTCCCAC‑TAACTCTCAGATTGACATgg                                                                                                                                                      >  1:348884/1‑301 (MQ=255)`  
                                                                                                                                                    `aCCGGAACCTCCGATGGTCAGTGTGGATTCGAGCCTGATGGTCGAGCCGAACTTGACGACCGAGATGCTAAACGTATTCTCGCAGTGACCCAGAAGGGTGACGCATGGATTCGTGCGTTACAGGATACTATTCGTGAAC‑TGCAACGTAAGCAGGAAATCAAGTAAGGAGGCAATGTGTCTACTCAATCCAAT‑CGTAATGCGCTCGTA‑GTGGCGCAACTGAAAGTAGACTTC‑GTGGCGTTCCTATTCGTCTTATGGAAGGCGCTAAACCTACCGGTGCCCAC‑TAAGTGTCAGATTGACATgg                                                                                                                                                      >  1:178298/1‑301 (MQ=255)`  
                                                                                                                                                     `ccGGAAACTCCGATGGTCAGTGTGGATTCGAGCCTGATGGTCGAGCCGAACTTGACGACCGAGATGCTAAACGTATTCTCGCAGTGACCCAGAAGGGTGACGCATGGATTCGTGCGTTACAGGATACTATTCGTGAAC‑TGCAACGTAAGCAGGAAATCAAGTAAGGAGGCAATGTGTCTACTCAATCCAAT‑CGTAATGCGCTCGTA‑GTGGCGCAACTGAAAGGAGACTTC‑GTGGCGTTCCTATTCGTCTTATGGAAGGCGCTAAACCTACCGGTGCCCAC‑TAAGTGTCAGATTGACATGGc                                                                                                                                                     <  1:587522/301‑1 (MQ=255)`  
                                                                                                                                                        `tgtgtataagagacaggtgtGGATTCGAGCCTGATGGTCGAGCCGAACTTGACGACCGAGATGCTAAACGTATTCTCGCAGTGACCCAGAAGGGTGACGCATGGATTCGTGCGTTACAGGATACTATTCGTGAAC‑TGCAACGTAAGCAGGAAATCAAGTAAGGAGGCAATGTGTCTACTCAATCCAAT‑CGTAATGCGCTCGTA‑GTGGCGCAACTGAAAGTAGACTTC‑GTGGCGTTCCTATTCGTCTTATGGAAGGCGCTAAACCTACCGGTGCCCAC‑TAAGTGTCAGATTGACATGGCTaa                                                                                                                                                  <  1:199714/285‑1 (MQ=255)`  
                                                                                                                                                          `aCCTCCGATGGTCAGTGTGGATTCGAGCCTGATGGTCGAGCCGAACTTGACGACCGAGATGCTAAACGTATTCTCGCAGTGACCCAGAATGGTGACGCATGGATTCGTGCGTTACAGGATACTATTCGTGAAC‑TGCAACGTAAGCAGGAAATCAAGTAAGGAGGCAATGTGTCTACTCAATCCAAT‑CGTAATGCGCTCGTA‑GTGGCGCAACTGAAATGAGACTTC‑GTGGCGTTCCTATTCGTCTTATGGAAGGCGCTAAACCTACCGGTGCCCAC‑TAAGTGTCAGATTGACATGGCTAAgg                                                                                                                                                <  1:266742/301‑1 (MQ=255)`  
                                                                                                                                                          `aCCTCCGATGGTCAGTGTGGATTCGAGCCTGATGGTCGAGCCGAACTTGACGACCGAGATGCTAAACGTATTCTCGCAGTGACCCAGAAGGGTGACGCATGGATTCGTGCGTTACAGGATACTATTCGTGAAC‑TGCAACGTAAGCAGGAAATCAAGTAAGGAGGCAATGTGTCTACTCAATCCAAT‑CGTAATGCGCTCGTA‑GTGGCGCAACTGAAAGGAGACTTC‑GTGGCGTTCCTATTCGTCTTATAGAAGGCGCTAAACCTACCGGTGCCCAC‑TAAGTGTCAGATTGACATGGCTAAgg                                                                                                                                                <  1:340685/301‑1 (MQ=255)`  
                                                                                                                                                            `cTCCGATGGTCAGTGTGGATTCGAGCCTGATGGTCGAGCCGAACTTGACGACCGAGATGCTAAACGTATTCTCGCAGTGACCCAGAAGGGTGACGCATGGATTCGTGCGTTACAGGATACTATTCGTGAAC‑TGCAACGTAAGCAGGAAATCAAGTAAGGAGGCAATGTGTCTACTCAATCCAAT‑CGTAATGCGCTCGTA‑GTGGCGCAACTGAAAGGAGACTTC‑GTGGCGTTCCTATTCGTCTTATGGAAGGCGCTAAACCTACCGGTGCCCAC‑TAAGTGTCAGAtctgtctcttatacaca                                                                                                                                              >  1:663055/1‑285 (MQ=255)`  
                                                                                                                                                                `gATGGTCAGTGTGGATTCGAGCCTGATGGTCGAGCCGAACTTGACGACAGAGATGCTAAACGTATTCTCGAAGTGACCCAGAAGGGTGACGCATGGATTCGTGCGTTACAGGATACTATTCGTGAAC‑TGCAACGTAAGCAGGAAATCAAGTAAGGAGGCAATGTGTCTACTCAATCCAAT‑CGTAATGCGCTCGTA‑GTGGCGAAACTGAAAGGAGACTTC‑GTGGCGTTCCTATTCGTCTTATGGAAGGCGCTAAACCTACCGGTGCCCAC‑TAAGTGTCAGATTGACATGGCTAAGGTACTgg                                                                                                                                          <  1:688267/301‑1 (MQ=255)`  
                                                                                                                                                                   `ggTCAGTGTGGATTCGAGCCTGATGGTCGAGCCGAACTTGACGACCGAGATGCTAAACGTATTCTCGCAGTGACCCAGAACGGTGACGCATGGATTCGTGCGTTACAGGATACTATTCGTGAAC‑TGCAACGTAAGCAAGAAATCAAGTAAGGAGGCAATGTGTCTACTCAATCCAAT‑CGTAATGCGCTCGTA‑GTGGCGCAACTGAAAGGAGACTTC‑GTGGCGTTCCTATTCGTCTTATGGAAGGCGCTAAACCTACCGGTGCCAAC‑TAAgtgctgtctcttatacacatctccgagcccac                                                                                                                                       >  1:322778/1‑272 (MQ=255)`  
                                                                                                                                                                      `cAGTGTGGATTCGAGCCTGATGGTCGAGCCGAACTTGACGACCGAGATGCTAAACGTATTCTCGCAGTGACCCAGAAGGGTGACGCATGGATTCGTGCGTTACAGGATACTATTCGTGAAC‑TGCAACGTAAGCAGGAAATCAAGTAAGGAGGCAATGTGAATACTCAATCCAAT‑CGTAATGCGCTCGTA‑GTGGCGCAACTGAAAGGAGACTTA‑GTGGCGTTCCTATTCGTCTTATGGAAGGCGCTAAACCTACCGGTACCCAC‑TACGTGTCAGATTGACATGGCTAAGGTGCTGGCGAAT‑g                                                                                                                                   >  1:353689/1‑301 (MQ=255)`  
                                                                                                                                                                       `aGTGTGGATTCGAGCCTGATGGTCGAGCCGAACTTGACGACCGAGATGCTAAACGTATTCTCGCAGTGACCCAGAAGGGTGACGCATGGATTCGTGCGTTACAGGATACTATTCGTGAAC‑TGCAACGTAAGCAGGAAATCAAGTAAGGAGGCAATGTGTCTACTCAATCCAAT‑CGTAATGCGCTCGTA‑GTGGCGCAACTGAAAGGAGACTTC‑GTGGCGTTCCTATTCGTCTTATGGAAGGCGCTAAACCTACCGGTGCCCAC‑TAAGTGTCAGATTGACATGGCTAAGGTGCTGGCGAAT‑gg                                                                                                                                  <  1:215458/301‑1 (MQ=255)`  
                                                                                                                                                                           `tgGATTCTAGCCTGATGGTCGAGCCGAACTTGACGACCGAGATGCTAAACGTATTCTCGCAGTGACCCAGAAGGGTGACGCATGGATTCGTGCGTTACAGGATACTATTCGTGAAC‑TGCAACGTAAGCAGGAAATCAAGTAAGGAGGCAATGTGTCTACTCAATCCAAT‑CGTAATGCGCTCGTA‑GTGGCGCAACTGAAAGGAGACTTC‑GTGGCGTTCCTATTCGTCTTATGGAAGGCGCTAAACTTACCGGTGCCCAC‑TAAGTGTCAGATTGACATGGCTAAGGTGCTGGCGAAT‑GGAGac                                                                                                                              <  1:111256/301‑1 (MQ=255)`  
                                                                                                                                                                           `tgGATTCGATCTTGATGGTCGAGCCGAACTTGACGACCGAGATGCTAAACGTATTCTCGCAGTGACCCAGAAGGGTGACGCATGGATTCGTGCGTTACAGGATACTATTCGTGAAC‑TGCAACGTAAGCAGGAAATCAAGTAAGGAGGCAATGTGTCTACTCAATCCAAT‑CGTAATGCGCTCGTA‑GTGGCGCAACTGAAATGAGACTTC‑GTGGCGTTCCTATTCGTCTTATGGAAGGCGCTAAACCTACCGGTGCCCAC‑TAAGTGTCAGATTGACATGGCTAAGGTGCTGGCGAAT‑GGAGac                                                                                                                              <  1:224592/301‑1 (MQ=255)`  
                                                                                                                                                                                          `tGGTCGAGCCGAACTTGACGACCGAGATGCTAAACGTATTCTCGCAGTGACCCAGAGGGGTAAGGCATGGTTCGGTCCGTAACAGG‑TTCTTTTTGTGAACTTGAACCGTAACCGGGAAA‑CAAATTAGGAGGGAATTTTGTTACTTAAACCAATCCGTATTG‑GCTCGGAGGGGGCGC‑ACTTGAAGGAGGCTTCTGTGCCG‑TCCTTTTCGTTTTTTTGGAGGCGGCTAACCTAACGGTTCCCACTTAATTTTCGAATTAAC‑TTGCTTAGGTGGTTGCGAATGGGAGAAAACAGGAATTTAAtt                                                                                                               >  1:694958/1‑300 (MQ=255)`  
                                                                                                                                                                                                `aGCCGAACTTGACGACCGAGATGCTAAACGTATTCTCGCAGTGACCCAGAAGGGTGACGCATGGATTCGTGCGTTACAGGATACTATTCGTGAAC‑TGCAACGTAAGCAGGAAATCAAGTAAGGAGGCAATGTGTCTACTCAATCCAAT‑CGTAATGCGCTCGTA‑GTGGCGCAACTGAAAGGAGACTTC‑GTGGCGTTCCTATTCGTCTTATGGAAGGCGCTAAACCTACCGGTGCCCAC‑TAAGTGTCAGATTGACATGGATAAGGTGCTGGCGAAT‑GGAGACAACAAGAAGTTCATCTTACAg                                                                                                         >  1:769460/1‑301 (MQ=255)`  
                                                                                                                                                                                                        `tataagagacagaTGCTAAACGTATTCTCGCAGTGACCAAGAAGGGTGACGCATGGATTCGTGCGTTACAGGATACTATTCGTGAAC‑TGCAACGTAAGCAGGAAATCAAGTAAGGAGGCAATGTGTCTACTCAATCCAAT‑CGTAATGCGCTCGTA‑GTGGCGCAACTGAAAGGAGACTTC‑GTGGCGTTCCTATTCGTCTTATGGAAGGCGCTAAACCTACCGGTGCCAAC‑TAAGTGTCAGATTGACATGGCTAAGGTGCTGGCGAAT‑GGAGAAAACAAGAAGTTCATCTTACAGGCTTGCCg                                                                                                 <  1:240291/291‑1 (MQ=255)`  
                                                                                                                                                                                                        `agatgtgtataagagacagaCGTATTCTCGCAGTGACCCAGAAGGGTGACGAATGGATTCGTGCGTTACAGGATACTATTCGTGAAC‑TGCAACGTAAGCAGGAAATCAAGTAAGGAGGCAATGTGTCTACTCAATCCAAT‑CGTAATGCGCTCGTA‑GTGGCGCAACTGAAAGGAGACTTC‑GTGGCGTTCCTATTCGTCTTATGGAAGGCGCTAAACCTACCGGTGCCCAC‑TAAGTGTCAGATTGACATGGCTAAGGTGCTGGCGAAT‑GGAGACAACAAGAAGTTCATCTTACAGGCTTTCCg                                                                                                 <  1:280000/282‑1 (MQ=255)`  
                                                                                                                                                                                                                  `agaTGCTAAACGTATTGTCGAAGGGACCCAGAAGGGTGACGCATGGATTCGTGCGTTACAGGATACTATTCGTGAAC‑TGCAACGTAAGCAGGAAATCAAGTAAGTAGGCAATGTGTCTACTCAATCCAAT‑CGTAATGCGCTCGTA‑GTGGCGCAACTGAAAGGAGACTTC‑GT‑GCGTTCCTATTCGTCTTATGGAAGGCGCTAAACCTACCGGTGCCCAC‑TAAGTGTCAGATTGACATGGCTAAGGTGTTGGCGAAT‑GGAGACAACAAGAAGTTCATCTTACAGGCTTTCCGTGGTATCGGTa                                                                                      <  1:412024/301‑1 (MQ=255)`  
                                                                                                                                                                                                                    `aTGCTAAACGTATTCTCGCAGTGACCCAGAAGGGTGACGCATGGATTCGTGCGTTACAGGATACTATTCGTGAAC‑TGCAACGTAAGCAGGAAATCAAGTAAGGAGGCAATGTGTCTACTCAATCCAAT‑CGTAATGCGCTCGTA‑GTGGCGCAACTGAAAGGAGACTTC‑GTGGCGTTCCTATTCGTCTTATGGAAGGCGCTAAACCTACCAGTGCCCAC‑TAAGTGTCAGATTGACATGGCTAAGGTGCTGGCGAAT‑GGAGACAACAAGAAGTTCATCTTACAGGCTTTCCGTGGTATCGGTaa                                                                                     >  1:491290/1‑301 (MQ=255)`  
                                                                                                                                                                                                                      `gCTAAACGTATTCTCGCAGTGACCCAGAATGGTGACGCATGGATTCGTGCGTTACAGGATACTATTCGTGAAA‑TGCAACGTAAGCAGGAAATCAAGTAAGGAGGCAATGTGTCTACTCAATGCAAT‑CGTAATGCGCTCGTA‑GTGGCGCAACTGAAAGGAGACTTC‑GCGGCGTTCCTATTCGTCTTATGGAAGGCGCTAAACCTACCGGTTCCCAC‑TAAGTGTCAGATTGACATGGCTAAGGTGCTGGCGAAT‑GGAGACAACAAGAAGTTCAGCTTACATGCTTTCCGTGGTATCGGTAAga                                                                                   >  1:757726/1‑300 (MQ=255)`  
                                                                                                                                                                                                                                `ttCTCGCAGTGACCCAGAAGGGTGACGCATGGATTCGTGCGTTACAGGATACTATTCGTGAAC‑TGCAACGTAAGCAGGAAATCAAGTAAGGAGGCAATGTGTCTACTCAATCCAAT‑CGTAATGCGATCGTA‑GTGGCGCAACTGAAAGGAGACTTC‑GTGGCGTTCCTATTCGTCTTATGGAAGGCGCTAAACCTACCGGTGCCAAC‑TAAGTGTCAGATTGACATGGCTAAGGTGCTGGCGAAT‑GGAGACAACAAGAAGTTCATCTTACAGGCTTTCCGTGGTATCTGtctcttatacacatc                                                                         >  1:458220/1‑287 (MQ=255)`  
                                                                                                                                                                                                                                `aaagagacaggACCTAGAAGGGTGACGCATGGATTCGTGCGTTACAGGATACTATTCGTGAAC‑TGCAACGTAAGCAGGAAATCAAGTAAGGAGGCAATGTGTCTACTCAATCCAAT‑CGTAATGCGCTCGTA‑GTGGCGCAACTGAAAGGAGACTTC‑GTGGCGTTCCTATTCGTCTTATGGAAGGCGCTAAACCTACCGGTGCCCAC‑TAAGTGTCAGATTGACATGGCTAAGGTGCTGGCGAAT‑GGAGACAACAAGAAGTTCATCTTACAGGCTTTCCGTGGTATCGGTAAGTCGTTCATcac                                                                         <  1:231353/291‑1 (MQ=255)`  
                                                                                                                                                                                                                                      `cAGTGACCCAGAAGGGTGACGCATGGATTCGTGCGTTACAGGATACTATTCGTGAAC‑TGCAACGTAAGCAGGAAATCAAGTAAGGAGGCAATGTGTCTACTCAATCCAAT‑CGTAATGCGCTCGTA‑GTGGCGCAACTGAAAGGAGACTTC‑GTGGCGTTCCTATTCGTATTATGGAAGGCGCTAAACCTACCGGTG‑CAAC‑TAAGTGTCAGATTGACATGGCTAAGGTGCTGGCGAAT‑GGAGACAACATGAAGTTCATCTTTCAGGCTTTCCGTGGTATCGGTAATTTGTTCACCACATGTGct                                                                  >  1:667538/1‑300 (MQ=255)`  
                                                                                                                                                                                                                                         `gagatgtgtataagagacagtGGATTCGTGCGTTACAGGATACTATTCGTGAAC‑TGCAACGTAAGCAGGAAATCAAGTAAGGAGGCAATGTGTCTACTCAATCCAAT‑CGTAATGCGATCGTA‑GTGGCGCAACTGAAAGGAGACTTC‑GTGGCGTTCCTATTCGTCTTATGGAAGGCGCTAAACCTACCGGTGCCCAC‑TAAGTGTCAGATTGACATGGCTAAGGTGCTGGCGAAT‑GGAGACAACAAGAAGTTCATCTTACAGGCTTTCCGTGGTATCGGTAAGTCGTTCATCACATGTGcgtt                                                                <  1:578069/281‑1 (MQ=255)`  
                                                                                                                                                                                                                                         `cggagatgtgtataagggacaggATTCGTGCGTTACAGGATACTATTCGTGAAC‑TGCAACGTAAGCAGAGAATCAAGTAAGGGAGCAATGTGTCTACTCAATCCAAT‑CGTAGTGCACTCGTA‑GTGGCGCAACTTAAAGGAGACTTC‑GTGGCGTTCCTATTCGTCTTATGGAAGGCGCTAAACCTACCGGTGCCCAC‑TAAGTGTCAGATTGACATGGCTAAGGTGCTGGCGAAT‑GGAGACAACAAGAAGTTCATCTTACAGGCTTTCCGTGGTATCGGTAAGTCGTTCATCACATGTGcgtt                                                                <  1:219611/280‑1 (MQ=255)`  
                                                                                                                                                                                                                                          `gACCCAGAAGGGTGACGCATGGATTCGTGCGTTACAGGATACTATTCGTGAAC‑TGCAACGTAAGCAGGAAATCAAGTAAGGAGGCAATGTGTCTACTCAATCCAAT‑CGTAATGCGCTCGTA‑GTGGCGCAACTGAAAGGAGACTTC‑GTGGCGTTCCTATTCGTCTTATGGAAGGCGCTAAACCTACCGGTGCCCAC‑TAAGTGTCAGATTGACATGGCTAAGGTGCTGGCGAAT‑GGAGACAACAAGAAGTTCATCTTACAGGCTTTCCGTGGTATCGGTAAGTCGTTCATcacactgtctctt                                                               >  1:26133/1‑292 (MQ=255)`  
                                                                                                                                                                                                                                          `gACCAAGAAGGGGGACGCATGGATTCGTGCGTTACAGGATACTATTCGTGAAC‑TGCAACGTAAGCAGGAAATCAAGTAAGGAGGCAATGTGTCTACTCAATCCAAT‑CGTAATGCGCTCGTA‑GTGGCGCAACTGAAAGGAGACTTC‑GTGGCGTTCCTATTCGTCTTATGGAAGGCGCTAAACCTACCGGTGCCCAC‑TAAGTGTCAGATTGACATGGCTAAGGTGCTGGCGAAT‑GGAGACAACAAGAAGTTCATCTTACAGGCTTTCCGTGGTATCGGTAAGTCGTTCATCACATGTGcgttc                                                               <  1:732977/301‑1 (MQ=255)`  
                                                                                                                                                                                                                                            `tcggagatgtgtataagagacagcGTGGGTTACAGGATACTATTCGTGAAC‑TGCAACGTAAGCAGGAAATCAAGTAAGGAGGCAATGTGTCTACTCAATCCAAT‑CGTAATGCGCTCGTA‑GTGGCGCAACTGAAAGGAGACTTC‑GTGGCGTTCCTATTCGTCTTATGGAAGGCGCTAAACCTACCGGTGCCCAC‑TAAGTGTCAGATTGACATGGCTAAGGTGCTGGCGAAT‑GGAGACAACAAGAAGTTCATCTTACAGGCTTTCCGTGGTATCGGTAAGTCGTTCATCACATGTGcattcgt                                                             <  1:328724/278‑1 (MQ=255)`  
                                                                                                                                                                                                                                            `cccAGAAGGGTGACGCATGGATTCGTGCGTTACATGATACTATTCGTGAAC‑TGCAACGTAAGCAGGAAATCAAGTAAGGAGGCAATGTGTCTACTCAATCCAAT‑CGTAATGCGCTCGTA‑GTGGCGCAACTGAAAGGAGACTTC‑GTGGCGTTCCTATTCGTCTTATGGAAGGCGCTAAACCTACCGGTGCCCAC‑TAAGTGTCAGATTGACATGGCTAAGGTGCTGGCGAAT‑GGAGACAACAAGAAGTTCATCTTACAGGCTTTCTGTGGTATCGGTAAGTCGTTCATCACCtgtctcttatt                                                             >  1:104739/1‑293 (MQ=255)`  
                                                                                                                                                                                                                                             `ccAGAAGGGTGACGCATGGATTCGTGCGTTACAGGATACTATTCGTGAAC‑TGCAACGTAAGCAGGAAATCAAGTAAGGAGGCAATGTGTCTACTCAATCCAAT‑CGTAATGCGCTCGTA‑GTGGCGCAACTGAAAGGAGACTTC‑GTGGCGTTCCTATTCGTCTTATGGAAGGCGCTAAACCTACCGGTGCCCAC‑TAAGTGTCAGATTGACATGGCTAAGGTGCTGGCGAAT‑GGAGACAACAAGAAGTTCATCTTACAGGCTTTCCGTGGTATCGGTAAGTCGTTCATCACATGTGcgttcgtt                                                            >  1:427591/1‑301 (MQ=255)`  
                                                                                                                                                                                                                                                   `cggagatgtgtataagagacagTTACAGGATACTATTCGTGAAC‑TGCAACGTAAGCAGGAAATCAAGTAAGGAGGCAATGTGTCTACTCAATCCAAT‑CGTAATGCGCTCGTA‑GTGGCGCAACTGAAAGGAGACTTC‑GTGGCGTTCCTATTCGTCTTATGGAAGGCGCTAAACCTACCGGTGCCCAC‑TAAGTGTCAGATTGACATGGCTAAGGTGCTGGCGAAT‑GGAGACAACAAGAAGTTCATCTTACAGGCTTTCCGTGGTATCGGTAAGTCATTCATCACATGTGCGTTCGTTGTGTgg                                                      <  1:44183/280‑1 (MQ=255)`  
                                                                                                                                                                                                                                                            `aTGGATTCGTGCGTTACAGGATACTATTCGTGAAC‑TGCAACGTAAGCAGGACATCAAGTAAGGAGTCAATTTGTCTACTCACTCCACT‑CGTCCTGCGCTCGTA‑GTGGCGAAACTGAAAGGAGACTTC‑TTCTCGTTCCTATTCGTCTTATGGAAGGCGCTAAACCTACCGGTGCACAC‑TAAGTGTCAGATTGACATGGCTAAGGTGCTGGCGAAT‑GGAGACAACAAGAAGTTCATCTTACAGGCTTTCAGTGGTATCGGTAAGTCGTTCATCACATGTGCGTTCGTTTTGTGGTCCTTATgg                                             >  1:609269/1‑301 (MQ=255)`  
                                                                                                                                                                                                                                                              `tcggagatgtgtataagagacagaTTCGTGAAC‑TGCAACGTAAGCAGGAAATCAAGTAAGGAGGCAATGTGTCTACTCAATCCAAT‑CGTAATGCGCTCGTA‑GTGGCGCAACTGAAAGGAGACTTC‑GTGGCGTTCCTATTCGTCTTATGGAAGGCGCTAAACCTACCGGTGCCCAC‑TAAGTGTCAGATTGACATGGCTAAGGTGCTGGCGAAT‑GGAGACAACAAGAAGTTCATCTTACAGGCTTTCCGTGGTATCGGTAAGTCGTTCATCACATGTGCGTTCGTTGTGTGGTCCTTATGgag                                           <  1:343692/278‑1 (MQ=255)`  
                                                                                                                                                                                                                                                              `ataagagacagTTACAGGATACTATTCGTGAAC‑TGTAACGTAAGCAGGTAATCAAGTAAGGAGGCAATGTGTTTACTCAAACCAAT‑CGTAATGCGCTCGTA‑GTGGCGCAACTGAAAGGAGACTTC‑GTGGCGTTCCTATTCGTCTTATGGAAGGCGCTAAACCTACCGGTGCCCAC‑TAAGTGTCAGATTGACATGGCTAAGGTGCTGGCGAAT‑GGAGACAACAAGAAGTTCATCTTACAGGCTTTCCGTGGTATCGGTAAGTCGTTCATCACATGTGCGTTCGTTGTGTGGTCCTTATGgag                                           <  1:783858/291‑1 (MQ=255)`  
                                                                                                                                                                                                                                                                `aTTCGTGCGTTACAGGATACTATTCGTGAAC‑TGCAACGTAAGCAGGAAATCAAGTAAGGAGGCAATGTGTCTACTCAATCCAAT‑CGTAATGCGCTCGTA‑GTGGCGCAACTGAAAGGAGACTTC‑GTGGCGTTCCTATTCGTCTTATGGAAGGCGCTAAACCTACCGGTGCCCAC‑TAAGTGTCAGATTGACATGGCTAAGGTACTGGCGAAT‑GGAGACAACAAGAAGTTCATCTTACAGGCTTTCCGTGGTATCGGTAAGTCGTTCATCACATGTGCGTTCGTTGTGTGGTCCTTATGgagag                                         <  1:88378/301‑1 (MQ=255)`  
                                                                                                                                                                                                                                                                 `ttCGTGCGTTACAGGATACTATTCGTGAAC‑TGCAACGTAAGCAGGAAATCAAGTAAGGAGGCAATGTGTCTACTCAATCCAAT‑CGTAATGCGCTCGTA‑GTGGCGCAACTGAAAGGAGACTTC‑GTGGCGTTCCTATTCGTCTTATGGAAGGCGCTAAACCTACCGGTGCCCAC‑TAAGTGTCAGATTGACATGGCTAAGGTGCTGGCGAAT‑GGAGACAACAAGAAGTTCATCTTACAGGCTTTCCGTGGTATCGGTAAGTCGTTCATCACATGTGCGTTCGTTGTGTGGTCCTTATGgagaga                                        >  1:82334/1‑301 (MQ=255)`  
                                                                                                                                                                                                                                                                    `gTGCGTTACAGGATACTATTCGTGAAC‑TGCAACGTAAGCAGGAAATCAAGTAAGGAGGCAATGTGTCTACTCAATCCAAT‑CGTAATGCGCTCGTA‑GTGGCGCAACTGAAAGTAGACTTC‑GTGGCGTTCCTATTCGTCTTATGGAAGGCGCTAAACCTACCGGTGCCCAC‑TAAGTGTCAGATTGACATGGCTAAGGTGCTGGCGAAT‑GGAGACAACAAGAAGTTCATCTTACAGGCTTTCCGTGGTATCGGTAAGTCGTTCATCACATGTGCGTTCGTTGTGTGGTCCTTATGGAGAGAccc                                     >  1:517167/1‑301 (MQ=255)`  
                                                                                                                                                                                                                                                                    `gTGCGTTACAGGATACTATTCGTGAAC‑TGCAACGTAAGCAGGAAATCAAGTAAGGAGGCAATGTGTCTACTCAATCCAAT‑CGTAATGCGCTCGTA‑GTGGCGCAACTGAAAGGAGACTTC‑GTGGCGTTCCTATTCGTCTTATGGAAGGCGCTAAACCTACCGGTGCCCAC‑TAAGTGTCAGATTGACATGGCTAAGGTGCTTGCGAAT‑GGAGACAACAAGAAGTTCATCTTACAGGCTTTCCGTGGTATCGGTAAGTCGTTCATCACATGTGCGTTCGTTGTGTggctgtctcttatacacat                                     >  1:95949/1‑284 (MQ=255)`  
                                                                                                                                                                                                                                                                    `gTGCGTTACAGGATACTATTCGTGAAC‑TGCAACGTAAGCAGGAAATCAAGTAAGGAGGCAATGTGTCTACTCAATCCAAT‑CGTAATGCGCTCGTA‑GTGGCGCAACTGAAAGGAGACTTC‑GTGGCGTTCCTATTCGTCTTATGGAAGGCGCTAAACCTACCGGTGCCCAC‑TAAGTGTCAGATTGACATGGCTAAGGTGCTGGCGAAT‑GGAGACAACAAGAAGTTCATCTTACAGGCTTTCCGTGGTATCGGTAAGTCGTTCATCACATTTGCGTTCGTTGTGTGGTCCTTATGGAGAGAccc                                     <  1:374342/301‑1 (MQ=255)`  
                                                                                                                                                                                                                                                                    `gTGCGGTACAGGGTACAAATCGTGACC‑TGGAACGTAAGTAGGTAAGCAAGTAAGGAGGCAACGGGTCTATGCAAGACAAT‑CGTCATGCGCTCGTA‑GTGGCGTAACTGAAAGGAGACCTC‑GTGGCCTTCCTATTCGACTTATGGAAGGCGCTAAATCGACCGGTGCTCAC‑TAAGTGTCAGATTTACATGGCTTAGGTGCTGGCGAAT‑GGAGACAACAAGAAGTTCATCTTACAGGCTTTCCGTGGTATCGGTAAGTCGTTCATCACATGTGCGTTCGTTGTGTGGTCCTTATGGAGAGAccc                                     <  1:483084/301‑1 (MQ=255)`  
                                                                                                                                                                                                                                                                         `ttACAGGATACTATTTGTGAAC‑TGCAACGTAAGCAGGAAATCAAGTAAGGAGGCAATGTGTCTACTCAATCCAAT‑CGTAATGCGCTCGTA‑GTGGCGCAACTGAAAGGAGACTTC‑GTGGCGTTCCTATTCGTCTTATGGAAGGCGCTAAACCTACCGGTGCCCAC‑TAAGTGTCAGATTGACATGGCTAAGGTGCTGGCGAAT‑GGAGACAACAAGAAGTTCATCTTACAGGCTTTCCGTGGTATCGGTAAGTCGTTCATCACATGTGCGTTCGTTGTGTGGTCCTTATGgagctgtctcttat                                >  1:726433/1‑290 (MQ=255)`  
                                                                                                                                                                                                                                                                          `tACAGGATACTATTCGTGAAC‑TGCAACGTAAGCAGGAAATCAAGTAAGGAGGCAATGTGTCTACTCAATCCAAT‑CGTAATGCGCTCGTA‑GTTGCGCAACTGAAAGGAGACTTC‑GTGGCGTTCCTATTCGTCTTATGGAAGGCGCTAAACCTACCGGTGCCCAC‑TAAGTGTCAGATTGACATGGCTAAGGTGCTGGCGAAT‑GGTGACAACAAGAAGTTCATCTTACAGGCTTTCCGTGGTATCGGTAAGTCGTTCATCACATGTGCGTTCGTTGTGTGGTCCTTATGGAGAGACCCTCAGtt                               <  1:398224/301‑1 (MQ=255)`  
                                                                                                                                                                                                                                                                           `aCAGGATACTATTCGTGAAC‑TGCAACGTAAGCAGGAAATCAAGTAAGGAGGCAATGTGTCTACTCAATCCAAT‑CGTAATGCGCTCGTA‑GTGGCGCAACTGAAAGGAGACTTC‑GTGGCGTTCCTATTCGTCTTATGGAGGGCGCTAAACATACCGGTGCCCAC‑TAAGTGTCAGATTGACATGGCTAAGGTGCTGGCGAAT‑GGAGACAACAAGAAGTTCATCTTACAGGCTTTCCGTGGTATCGGTAAGTCGTTCATCACATGTGCGTTCGTTGTGTggctgtctcttatacacatctccgag                              >  1:336985/1‑277 (MQ=255)`  
                                                                                                                                                                                                                                                                           `aCAGGATACTATTCGTGAAC‑TGCAACGTAAGCAGGAAATCAAGTAAGGAGGCAATGTGTCTACTCAATCCAAT‑CGTAATGCGCTCGTA‑GTGGCGCAACTGAAAGGAGACTTC‑GTGGCGTTCCTATTCGTCTTATGGAAGGCGCTAAACCTACCGGTGCCCAC‑TAAGTGTCAGATTGACATGGCTAAGGTGCTGGCGAAT‑GGAGACAACAAGAAGTTCATCTTACAGGCTTTCCGTGGTATCGGTAAGTCGTTCATCACATGTGCGTTCGTTGTGTGGTCCTTATGgagctgtctcttatac                              >  1:477482/1‑288 (MQ=255)`  
                                                                                                                                                                                                                                                                           `aCAGGATACTATTCGTGAAC‑TGCAACGTAAGCAGGAAATCAAGTAAGGAGGCAATGTGTCTACTCAATCCAAT‑CGTAATGCGCTCGTA‑GTGGCGCAACTGAAAGGAGACTTC‑GTGGCGTTCCTATTCGTCTTATGGAAGGCGCTAAACCTACCGGTGCCCAC‑TAAGTGTCAGATTGACATGGCTAAGGTGCTGGCGAAT‑GGAGACAACAAGAAGTTCATCTTACAGGCTTTCCGTGGTATCGGTAAGTCGTTCATCACATGTGCGTTCGTTGTGTGGTCCTTATGGAGAGACCCTCAGTTg                              >  1:38620/1‑301 (MQ=255)`  
                                                                                                                                                                                                                                                                           `aCAGGATACTATTCGTGAAC‑TGCAACGTAAGCAGGAAATCAAGTAAGGAGGCAATGTGTCTACACAATCCAAT‑CGTAATGCGCTCGTA‑GTGGCGCAACTGAAAGGAGACTTC‑GTGGCGTTCCTATTCGTCTTATGGAAGGCGCTAAACCTACCGGTGCCCAC‑TAAGTGTCAGATTGACATGGCTAAGGTGCTGGCGAAT‑GGAGACAACAAGAAGTTCATCTTACAGGCTTTCCGTGGTATCGGTAAGTCGTTCATCACATGTGCGTTCGTTGTGTGGTCCTTATGGAGAGACCTTCAGttt                              >  1:800761/1‑300 (MQ=255)`  
                                                                                                                                                                                                                                                                              `gtaTACTATTCGTGAAC‑TGCAACGTAAGCAGGAAATCAAGTAAGGAGGCAATGTGTCTACTCAATCCAAT‑CGTAATGCGCTCGTA‑GTGGCGCAACTGAAAGGAGACTTC‑GTGGCGTTCCTATTCGTCTTATGGAAGGCGCTAAACCTACCGGTGCCCAC‑TAAGTGTAAGATTGACATGGCTAAGGTGCTGGCGAAT‑GGAGACAACAAGAAGTTCATCTTACAGGCTTTCCGTGGTATCGGTAAGTCGTTCATCACATGTGCGTTCGTTGTGTGGTCCTTATGGAGAGACCCTCAGTTGAAg                           >  1:721211/3‑301 (MQ=255)`  
                                                                                                                                                                                                                                                                              `ggATACTATTCGTGAAC‑TGCAACGTAAGCAGGAAATCAAGTAAGGAGGCAATGTGTCTACTCAATCCAAT‑CGTAATTCGCTCGTA‑GTGGCGCAACTGAAAGGAGACTTC‑GTGGCGTTCCTATTCGTCTTATGGAAGGCGCTAAACCTACCGGTGCCCAC‑TAAGTGTCAGATTGACATGGCTAAGGTGCTTGCGAAT‑GGAGACAACAAGAAGTTCATCTTACAGGCTTTCCGTGGTATCGGTAAGTCGTTCATCACATGTGCGTTCGTTGTGTGGTCCTTATggctgtctcttatacacatt                           >  1:276307/1‑283 (MQ=255)`  
                                                                                                                                                                                                                                                                              `ggATACTATTCGTGAAC‑TGCAACGTAAGCAGGAAATCAAGTAAGGAGGCAATGTGTCTACTCAATCCAAT‑CGTAATGCGCTCGTA‑GTGGCGCAACTGAAAGGAGACTTC‑GTGGCGTTCCTATTCGTCTTATGGAAGGCGCTAAACCTACCGGTTCCCAC‑TAAGTGTCAGATTGACATGGCTAAGGTGCTGGCGAAT‑GTAGACAACAAGAAGTTCATCTTACAGGCTTTCCGTGGTATCGGTAAGTCGTTCATCACATGTGCGTTCGTTGTGTGGTCCTTATGGAGAGACCATCAGTTGAAg                           <  1:45967/301‑1 (MQ=255)`  
                                                                                                                                                                                                                                                                              `ggATACTATTCGTGAAC‑TGCAACGTAAGCAGGAAATCAAGTAAGGAGGCAATGTGTCTACTCAATCCAAT‑CGTAATGCGCTCGTA‑GTGGCGCAACTGAAAGGAGACTTC‑GTGGCGTTCCTATTCGTCTTATGGAAGGCGCTAAACCTACCGGTGCCCAC‑TAAGTGTCAGATTGACATGGCTAAGGTGCTGGCGAAT‑GGAGACAACAAGAAGTTCATCTTACAGGCTTTCCGTGGTATCGGTAAGTCGTTCATCACATGTGCGTTCGTtgtgctgtctcttatacacatctccgagcccacg                           >  1:631146/1‑271 (MQ=255)`  
                                                                                                                                                                                                                                                                              `ggATACTATTCGTGAAC‑TGCAACGTAAGCAGGAAATCAAGTAAGGAGGCAATGTGTCTACTCAATCCAAT‑CGTAATGCGCTCGTA‑GTGGCGCAACTGAAAGGAGACTTC‑GTGGCGTTCCTATTCGTCTTATGGAAGGCGCTAAACCTACCGGTGCCCAC‑TAAGTGTCAGATTGACATGGCTAAGGTGCTGGCGAAT‑GGAGACAACAAGAAGTTCATCTTACAGGCTTTCCGTGGTATCGGTAAGTCGTTCATCACATGTGCGTTCGTTGTGTGGTCCTTATGGAGAGACCCTCAGTTGAAg                           >  1:654862/1‑301 (MQ=255)`  
                                                                                                                                                                                                                                                                                 `agatgtgtataagagacagaCGTAATCAGGAAATCAAGTAAGGAGGCAATGTGTCTACTCAATCCAAT‑CGTAATGCGCTCGTA‑GTGGCGCAACTGAAAGGAGACTTC‑GTGGCGTTCCTATTCGTCTTATGGAAGGCGCTAAACCTACCGGTGCCCAC‑TAAGTGTCAGATTGACATGGCTAAGGTGCTGGCGAAT‑GGAGACAACAAGAAGTTCATCTTACAGGCTTTCCGTGGTATCGGTAAGTCGTTCATCACATGTGCGTTCGTTGTGTGGTCCTTATGGAGAGACCCTCAGTTGAAGAt                         <  1:36904/282‑1 (MQ=255)`  
                                                                                                                                                                                                                                                                                  `aCTATTCGTGAAC‑TGCAACGTAAGCAGGAAATCAAGTAAGGAGGCAATGTGTCTACTCAATCCAAT‑CGTAATGCGCTCGTA‑GTGGCGCAACTGAAAGGAGACTTC‑GTGGCGTTCCTATTCGTCTTATGGAAGGCGCTAAACCTACCGGTGCCCAC‑TAAGTGTCAGATTGACATGGCTAAGGTGCTGGCGAAT‑TGAGACAACAAGAAGTTCATCTTACAGGCTTTCCGTGGTATCGGTAAGTCGTTCATCACATGTGCGTTCGTTGTGTGGTCCTTATggctgtctcttatacacatctccg                       >  1:749417/1‑279 (MQ=255)`  
                                                                                                                                                                                                                                                                                     `aTTCGTGAAC‑TGCAACGTAAGCAGGAAATCAAGTAAGGAGGCAATGTGTCTACTCAATCCAAT‑CGTAATGCGCTCGTA‑GTGGCGCAACTGAAAGGAGACTTC‑GTGGCGTTCCTATTCGTCTTATGGAAGGCGCTAAACCTACCGGTGCCCAC‑TAAGTGTCAGATTGACATGGCTAAGGTGCTGGCGAAT‑GGAGACAACAAGAAGTTCATCTTACAGGCTTTCCGTGGTATCGGTAAGTCGTTCATAACATGTGCGTTCGTTGTGTGGTCCTTATGGAGAGACCCTCAGTTGAAGATACttt                    >  1:593658/1‑300 (MQ=255)`  
                                                                                                                                                                                                                                                                                       `tCGTGAAC‑TGCAACGTAAGCAGGAAATCAAGTAAGGAGGCAATGTGTCTACTCAATCCAAT‑CGTAATGCGCTCGTA‑GTGGCGCAACTGAAAGGAGAATTC‑GTGGCGTTCCTATTCGTCTTATGGAAGGCGCTAAACCTACCGGTGCACAC‑TAAGTGTCAGATTGACATGGCTAAGGTGCTGGCGAAT‑GGAGACAACAAGAAGTTCATCTTACAGGCTTTCCGTGGTATCGGTAAGTCGTTCATCACATGTGCGTTCGTCGTGTGGTCCTTATGGAGAGACCCTCAGTTGAAGATACTTATc                  <  1:175700/301‑1 (MQ=255)`  
                                                                                                                                                                                                                                                                                           `gAAC‑TGCAACGTAAGCAGGAAATCAAGTAAGGAGGCAATGTGTCTACTCAATCCAAT‑CGTAATGCGCTCGTA‑GTGGCGCAACTTAAAGGAGACTTC‑GTGGCGTTCCTATTCGTCTTATGGAAGGCGCTAAACCTACCGGTGCCCAC‑TAAGTGTCAGATTGACATGGCTAAGGTGCTGGCGAAT‑GGAGACAACAAGAAGTTCATCTTACAGGCTTTCCGTGGTATCGGTAAGTCGTTCATCACATGTGCGTTCGTTGTGTGGTCCTTATGGAGAGACCCTCAGTTGAAGATACTTATCGTAt              >  1:446220/1‑301 (MQ=255)`  
                                                                                                                                                                                                                                                                                            `aaC‑TGCAACGTAAGCAGGAAATCAAGTAAGGAGGCAATGTGTCGACTCAATCCAAT‑CGTAATGCGCTCGTA‑GTGGCGCAACTGAAAGGAGACTTC‑GTGGCGTTCCTATTCGTCTTATGGAAGGCGCTAAACCTACCAGTGCCCAC‑TAAGTGTCAGATTGACATGGCTAAGGTGCTGGCGAAT‑GGAGACAACAAGAAGTTCATCTTACAGGCTTTCCGTGGTATCGGTAAGTCGTTCATCACATGTGCGTTCGTTGTGTGGTCCTTATGGAGAGACCCTCAGTTGAAGATACTTATCATATc             <  1:744055/301‑1 (MQ=255)`  
                                                                                                                                                                                                                                                                                                    `aCGTAAGCAGGAAATCAAGTAAGTAGGCAATGTGTCTACTCAATCCAAT‑CGTAATGCGCTCGTA‑GTGGCGCAACTGAAAGGGGACTTC‑GTGGCGTTCCTATTCGTCTTATGGAAGGCGCTAAACCTACCGGTGCCCAC‑TAAGTGTCAGATTGACATGGCTAAGGTGCTGGCGAAT‑GGAGACAACAAGAAGTTCATCTTACAGGCTTTCCGTGGTATCGGTAAGTCGTTCATCACATGTGCGTTCGTTGTGTGGTCCTTATGGAGAGACCCTCAGTTGAAGATACTTATCGTATCAGCctct      >  1:602663/1‑301 (MQ=255)`  

                                                                                                                                                                                                                                                                                                           `|`                                                                                                                                                                                                                                                                                                                
`AGTTGAGGCTGCGAAGAGCACTCAAAGAGCAATCGATGCGGTATCTGCTAAGTATCAAGAAGACCTTGCCGCGCTGGAAGGGAGCACTGATAGGATTATTTCTGATTTGCGTAGCGACAATAAGCGGTTGCGCGTCAGAGTCAAAACTACCGGAACCTCCGATGGTCAGTGTGGATTCGAGCCTGATGGTCGAGCCGAACTTGACGACCGAGATGCTAAACGTATTCTCGCAGTGACCCAGAAGGGTGACGCATGGATTCGTGCGTTACAGGATACTATTCGTGAAC‑TGCAACGTAAGTAGGAAATCAAGTAAGGAGGCAATGTGTCTACTCAATCCAAT‑CGTAATGCGCTCGTA‑GTGGCGCAACTGAAAGGAGACTTC‑GTGGCGTTCCTATTCGTCTTATGGAAGGCGCTAAACCTACCGGTGCCCAC‑TAAGTGTCAGATTGACATGGCTAAGGTGCTGGCGAAT‑GGAGACAACAAGAAGTTCATCTTACAGGCTTTCCGTGGTATCGGTAAGTCGTTCATCACATGTGCGTTCGTTGTGTGGTCCTTATGGAGAGACCCTCAGTTGAAGATACTTATCGTATCAGCCTCTAAGG  >  NC_001604.1/37048‑37643`

  

Alignment Legend

Aligned base mismatch/match (shaded by quality score): `ATCG/ATCG` `< 3 ≤` `ATCG/ATCG` `< 11 ≤` `ATCG/ATCG` `< 26 ≤` `ATCG/ATCG` `< 37 ≤` `ATCG/ATCG` `< 38 ≤` `ATCG/ATCG`

Unaligned base: `atcg`    Masked matching base: `atcg`    Alignment gap: `‑`    Deleted base: `‑`
